# A novel tetra-primer ARMS-PCR for genotyping of the *OPRM1* gene rs1799971 variant associated with opioid use disorders

**DOI:** 10.1186/s13104-023-06578-7

**Published:** 2023-11-14

**Authors:** P. J. Wijekumar, N. D.K. Ranadeva, A. R. Jayamaha, H. M.N.D.M. Herath, N. Noorden, S. S.N. Fernando

**Affiliations:** 1 KIU, Battaramulla, Sri Lanka; 2 Faculty of Medical Sciences, University of Sri Jayewardenepura, Sri Jayewardenepura, Sri Lanka

**Keywords:** Opioid use disorder, *OPRM1*, T-ARMS PCR, rs1799971

## Abstract

**Objectives:**

A SNV is a single nucleotide change that can occur at any point in the genome. SNVs are the most common genetic variants that occur in the human genome, and a number of SNVs have been found to be associated with human traits and disease. Researchers genotype SNVs using TaqMan technology, DNA microarray, MALDI-TOF mass spectrometry, and automated sequencing, which are expensive and time-consuming. The *OPRM1* gene rs1799971 (A118G) has been identified for its association with Opioid use disorder (OUD). The present study focused on developing a single step identification test using Tetra-Primer Amplification Refractory Mutation System-PCR (T-ARMS-PCR) to detect the presence of SNV *OPRM1* rs1799971 (A118G). This study was performed to optimize the protocol for the designed four primers and validate it using a total of 52 buccal samples from volunteers who are currently under rehabilitation for the drug abuse disorder.

**Results:**

Utilizing 52 DNA samples, a novel T-ARMS-PCR assay was successfully developed, tested, and validated. The products of the T-ARMS PCR for rs1799971 contained 395 bp as the control band, 186 bp as G allele (variant) and 257 bp as A allele (wild type), which were observed in the gel image. The genotype frequencies for the *OPRM1* gene rs1799971 (A118G) were 44% (22/52) of homozygous variant type (GG), 28.9% (15/52) of homozygous wild type (AA) and 28.9% (15/22) of heterozygous (AG). The G allele frequency was 56.7% and A allele frequency was 43.3%.

**Supplementary Information:**

The online version contains supplementary material available at 10.1186/s13104-023-06578-7.

## Introduction

Drug addiction is a complex disorder influenced by a variety of factors, including genetics. There is a 40 to 60% genetic contribution to the development of drug addiction [1–3]. Drug-related phenotypes are common, complex, and highly heritable traits. On the basis of twin and family studies, estimates of OUD risk heritability range from 23 to 54% [2, 4]. In recent years, candidate gene (CGAS) and genome-wide association studies (GWAS) have identified a vast number of single nucleotide variants (SNVs) associated with drug use, abuse, or dependence, most of which are related to opioid usage [5]. However, few genes associated with addiction risk or protection have been validated, and the genetic architecture of drug addiction remains obscure.

Most opioids, such as morphine and morphine derivatives, exert their analgesic effects through the human µ-opioid receptor (MOR). Additionally, endogenous opioid peptides such as enkephalins, endorphins, and dynorphins exert a broad range of physiological and behavioral effects via the MOR, including effects on pain perception, mood, motor control, and autonomic functions. At least in part, it is believed that genetic variation in the functionality or density of the µ-opioid receptor contributes to the substantial phenotypic differences in opioid response. MOR is encoded by the *OPRM1* gene. At present, the most prevalent SNV of the *OPRM1* gene is rs1799971 which is an A118G substitution. The rs1799971(G) allele in exon 1 of the µ- opioid receptor *OPRM1* gene causes asparagine (Asn) to be replaced by aspartic acid at residue 40 (Asp). This SNV has also been linked to differences in dopaminergic sensitivity during acute alcohol withdrawal and altered β-endorphin receptor binding capacity [6].

To date, *OPRM1* gene variants have been identified via PCR amplification of the appropriate gene fragment followed by automated DNA sequencing or PCR-RFLP (polymerase chain reaction-restriction fragment length polymorphism) [7, 8]. RFLP procedure is time-consuming, and, as a result, is unsuitable for rapid patient screening. Using Tetra-ARMS-PCR, we developed a rapid and reliable method for detecting the SNV at position rs1799971 of the human MOR gene. This technique has been previously utilized successfully for the detection of other SNVs in the human genome.

## Main text

## Materials and methods

The research was carried out at KIU’s Biomedical Science Laboratory in Sri Lanka. In this investigation in which a novel assay for the *OPRM1* gene rs1799971 (G) variant was developed and validated using a cohort of drug users (n = 52) in rehabilitation centers.

### Clinical sample collection and DNA extraction

Buccal swab samples were obtained from volunteers at selected rehabilitation centers in Sri Lanka. The participants were instructed to wash their mouths with clean water before the sample collection procedure. The buccal cells were collected using a swab. The swab was rubbed against the inside of the mouth several times on both sides. The swab was sealed in a separate container and kept on ice until transported to the laboratory.

From the buccal swab, DNA was extracted using the QIAamp DNA Mini kit (CAT: 51,304, Qiagen, Germany). The extracted DNA specimens were stored at -20^0^ C until genotyped.

### Designing primers

*OPRM1* gene rs1799971 (G) gene sequence was obtained from the NCBI (NCBI Reference Sequence: NC_000006.12). The 1000 bases with the SNV in the center from 5′ to 3′ direction of the DNA sequence was obtained from Serial Cloner which included the mutation in the center. A web-based tool, Primer 1 software, http://primer1.soton.ac.uk/primer1.html was utilized to generate the primer for identification of *OPRM1* gene rs1799971 (G) variant. Primer 1 software is specially developed for creating T-ARMS PCR primers [9]. The DNA sequence from the serial cloner was inserted into the Primer 1 software. The standard parameters to create T-ARMS primers were considered such as Tm, %GC, length and complementarity and product sizes [10]. The melting temperature of each primer was considered to select the final set of primers. NCBI primer blast, http://www.ncbi.nlm.nih.gov/tools/primer-blast/ was used to check the specificity of the primers and the primer details are illustrated in Table 1.

**Table 1:** Tetra-primer ARMS-PCR primers for the detection of *OPRM1* gene rs1799971 variant

Primers	Sequence (5’ to 3’)	Length (bp)	Tm (manufacturer) (^o^C)	Product Size (bp)
Forward inner primer (G allele)	GGGTCAACTTGTCCCACTTAGATGTCG	27	66.2	Control: 395 (G): 186 (A): 257
Reverse inner primer (A allele)	ACCGCATGGGTCGGACAGTTT	21	66.6	
Forward outer primer (5’ − 3’)	GTAAGAAACAGCAGGAGCTGTGGCAG	26	65.7	
Reverse outer primer (5’ − 3’)	CATGACCAGGAAGTTTCCGAAGAGC	25	65.0	

**Fig. 1:**
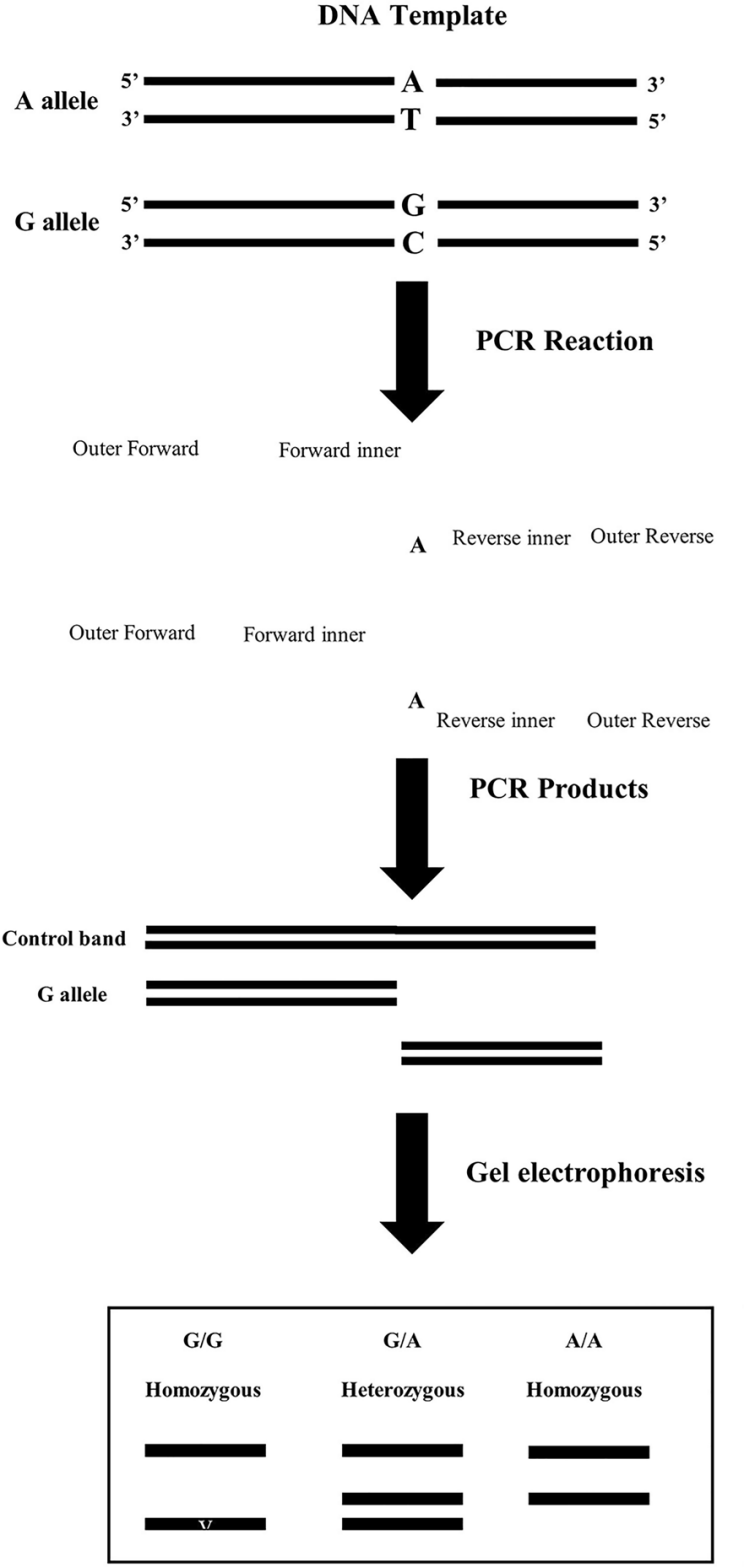
A layout of the T-ARMS PCR assay for *OPRM1* gene 1799971 variations

### **ARMS-PCR for genotyping of the*OPRM1*gene rs1799971**.

Gradient PCR was performed on the Thermal Cycler (Bio Rad) for the targeted variation *OPRM1* gene rs1799971. In the first attempt at PCR optimization, the manufacturer’s recommended primer annealing temperatures were used for the annealing temperature gradient. The final optimal cycle conditions were obtained after a series of optimization attempts. The final optimized protocol for this variant consisted of an initial denaturation of 3 min at 94 °C (initial denaturation), followed by 30 PCR cycles of 1 min at 94 °C (denaturation), 30 s at 58 °C (annealing temperature), 1 min at 72 °C and the final extension of 5 min at 72 °C. The optimized PCR master mixture contained 2 µl of the DNA extracted by the QIAamp DNA Mini kit, 5.0 µL of 5 × Green Go-Taq Flexi Buffer, 1.5 µL of MgCl_2_ (25 mM), 1.0 µL dNTP mixture (2.5 mM), 1.5 µL of 25µM of two outer primers, 1.0 µL of 25µM of two inner primers, 0.2 µL of 5U/UL Taq polymerase and 10.3 µl of dH_2_O.

### Gel Electrophoresis

The resultant PCR product of 10 µL were analyzed by 2% agarose gel electrophoresis, in TBE 1X buffer, containing ethidium bromide at 100 V for 45 min and visualized under a UV illuminator.

### Validation of genotyping

The sensitivity and specificity of the T-ARMS PCR was tested using 52 DNA samples from the volunteers using the developed protocol.

### Data Analysis

The genotype frequencies were calculated using data from the gel images (allele counts). The observed band sizes for homozygous wild type (AA) were at 186 bp, heterozygous variant (AG) were at 186 and 257 bp, and homozygous variant (GG) were at 25 bp. The allele frequencies for A (wild type) and G (variant) alleles, were calculated and tested for Hardy Weinberg equilibrium using an online calculator (available at: https://wpcalc.com/en/equilibrium-hardy-weinberg/).

## Results and discussion

**Fig. 2:**
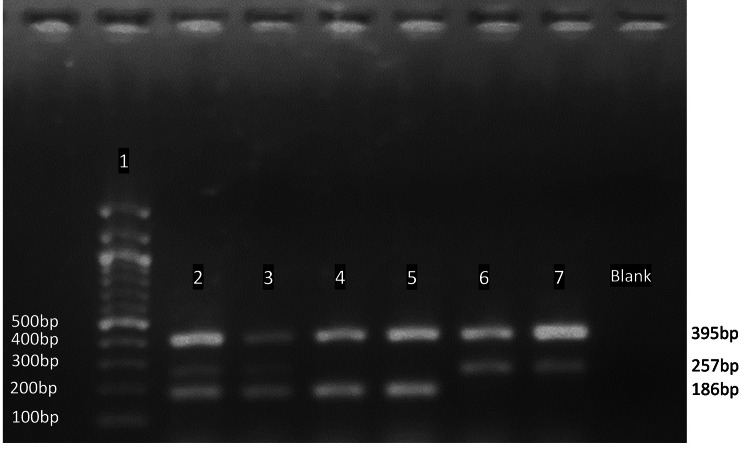
Gel band patterns of *OPRM1* gene rs1799971 (A118G). The Gel image depicts the T-ARMS-PCR genotyping results of the *OPRM1* mutation. The genotyping results shows 100 bp ladder (Lane 1), control band at 395 bp (Lane 2,3,4,5,6,7), variant G allele at 186 bp (Lane 2,3,4,5) and wild A allele at 257 bp (Lane 2,3,6,7). Samples in Lane 2, 3 are A/G, Lane 4, 5 are GG and Lane 6,7 are AA.

**Table 2:** Genotype and allele frequency of *OPRM1* gene rs1799971 variant

Samples (n = 52)	Genotype frequency			Allele frequency	
	AA	AG	GG	A (wild)	G (variant)
Opioid addict volunteers	15 (28.9%)	15 (28.9%)	22 (44%)	0.4327 (43.3%)	0.5673 (56.7%)

Given the importance of genetic variation in human disease, particularly in substance use disorders like OUD, important goals of genetic research is to uncover genetic variants associated with a particular disease. Since the completion of the Human Genome project, SNVs as genetic markers have been widely studied in different aspects of disorders. Researchers genotype SNVs using high-through put techniques such as the TaqMan technology, DNA microarray, MALDI-TOF mass spectrometry, and sequencing [11–15]. However, these techniques are expensive when applied to genotype a large population. The most commonly used approach for SNV genotyping is polymerase chain reaction (PCR) [16]. There are several modified PCR techniques available; traditionally, PCR followed by RFLP, was used to study SNVs related to OUD [8, 17]. However, due to the cost and time consumption of these techniques, there are various techniques that are validated. One such method, T-ARMS PCR, a simple and cost-effective SNV genotyping approach only involving single PCR followed by gel electrophoresis, was used to genotype the most common genetic variant of OUD, *OPRM1* gene rs1799971 variant. Extensive research has been conducted to investigate the potential significance of the *OPRM1* gene to an individual’s susceptibility to addiction. The most common SNV discovered in *OPRM1* is A118G, which is located in exon I and causes Asn40Asp amino acid substitution [19].

To determine the genotype, the tetra-primer ARMS-PCR employs four primers in a single PCR. Two non-allele-specific primers amplify the area containing the SNV at the start of the reaction (outer primers). The outer primers fragment serves as a template for the two allele-specific primers (inner primers) that will produce the allele-specific fragments. The two allele specific fragments can be identified in an agarose gel by placing the outer primers at different ranges from the variant nucleotide [10, 18]. However, the optimization procedure can be time-consuming and laborious [19] and a common limitation with the T-ARMS procedure is that the concentrations of MgCl_2_, four primers, dNTPs and annealing temperature need to be optimized in order to detect the DNA band accurately [19]. The protocol development was divided into three stages. The first stage was to carry out the initial trial of T-ARMS PCR using information from the literature to identify the bands. Then with the results of the initial protocol, the optimization procedure of MgCl_2_ was carried out. Different concentrations were used and ideally the 1.5 µL of 25mM was used for the protocol. Next, the optimization of the primers was carried out. One important step was balancing the concentrations of four primers and optimizing it. The outer primers reacted strongly to small changes of concentrations, and it was crucial for the inner primers to bind specifically to prevent formation of non-specific bands. Another challenge identified was the formation of non-specific bands at the bottom of the gel. To overcome this issue, PCR touchdown and several concentrations of four primers were used [20]. Finally, a lower volume of inner primers and higher volumes of outer primers with the same concentration produced a clear and specific band. A higher volume of outer primers inhibited the non-specific band formation. The final primer concentrations for rs1799971 detection are shown in Table 1.

The crucial step was to identify the optimum annealing temperature and different temperature gradients were carried out to optimize the annealing temperature. The gradient reaction was repeated till the heterozygous and homozygous controls were visible. It was observed that the gradient range was between 56^o^C to 66^o^C for all three bands. However, the DNA bands were most intense, thus the optimal annealing temperature was chosen as 58^o^C.

Furthermore, the non-specific band formation was due to the annealing temperature being different from the T_m_ of the four primers. In addition to this, the gel concentration was optimized for the visualization of the amplicons and a 2% gel was found to be optimal. The products of the T-ARMS PCR for rs1799971 contained 395 bp as the control band, 186 bp as G allele (variant) and 257 bp as A allele (wild type). A significant difference between the band sizes of the two alleles allowed visualization of the bands clearly [20]. Figure 1 shows the expected gel patterns for the protocol and Fig. 2 shows the actual image of the genotype results.

After validating the novel protocol, the final protocol was used to genotype 52 DNA samples of volunteers randomly selected from rehabilitation centers in Sri Lanka. All the volunteers in the sample cohort that were genotyped were males with a history of opioid usages. The expected gel band patterns were observed in the gel image results of the genotyped samples. The genotype and the allele frequencies of the samples are shown in Table 2. Out of the 52 samples genotyped, 44% were homozygous variants (GG), 28.9% were homozygous wild type (AA) and 28.9% were heterozygous (AG). The predominant genotype was GG (44%) and the minor allele frequency was 56.7% as shown in Table 2.

The *OPRM1* gene rs1799971 variant has been studied in various population and the present study add to the South Asian population genotyping data. The genotyping results were comparable to the studies conducted in the South Asian region as well as a study conducted in Sri Lanka by Dissabandara et al., [17]. The study concluded that 44% of volunteers were homozygous for the *OPRM1* gene rs1799971 variant, thus the present study is in line with the previous study.

## Limitations

- During the optimization procedure determining the DNA quality, correct concentrations of primers and PCR reagents were challenging since four primers should be optimized immediately with minimal primer dimer formation.
- A good DNA quality should be maintained with a minimum of 20ng/ml for a well optimized PCR protocol and high yield of the product.
- Buccal swab samples should be collected accurately to obtain a good quality DNA sample to obtain a good PCR.
- A random set of genotyped samples was not Sanger sequenced due to the limited time available and the funds available at the end of the project.

## Conclusion

Several methods for genotyping single nucleotide variants (SNVs) are available as due to the rapid development of molecular techniques. Nonetheless, the search for simple, robust, and less expensive techniques continues.

The objective of the study was to develop a T-ARMS-PCR based technique for genotyping the rs1799971 SNV in *OPRM1* genes in order establish an association between specific genetic variants and the phenotypic trait, assuming phenotypic data is available. An associated SNV could be used to expedite identification of an individual’s susceptibility to opioids and to design a personalized therapy. Tetra ARMS-PCR is a cost effective, efficient, and reproducible technique that could be utilized to genotype individuals with drug addiction based on their genetic variant-mutation.

### Electronic supplementary material

Below is the link to the electronic supplementary material.

Supplementary Material 1: Figure S1 - Gel bands of patient samples from 1 to 4. Figure S2 – Gel bands of patient samples from 5 to 21 with known A/G and G/G bands. Figure S3 – Gel bands of patient samples from 22 to 38 with known A/G and G/G bands. Figure S4 – Gel bands of patient samples from 39 to 52 with known A/G and G/G bands

Supplementary Material 2: Table S1 - Genotyping results of the 52 volunteer patients.

## Data Availability

All the datasets used and or analyzed during the current study are available from the corresponding author on reasonable request at jalini@kiu.ac.lk.

## Abbreviations

SNV: Single Nucleotide Variant
DNA: Deoxyribonucleic acid
MALDI: TOF–Matrix–assisted laser desorption/ionization–time of flight
OUD: Opioid Use Disorder
T-ARMS PCR: tetra–primer amplification refractory mutation system–polymerase chain
PCR: Polymerase chain reaction
CGAS: candidate gene association studies
GWAS: genome–wide association studies
MOR: µ–opioid receptor
PCR: RFLP–Polymerase chain reaction–restriction fragment length polymorphism
TBE: Tris/Borate/EDTA buffer
UV: Ultraviolet
Tm: Melting Temperature
MgCl2: Magnesium Chloride
dNTPs: deoxynucleotide triphosphates
