# Supplementary material for: A novel tetra-primer ARMS-PCR for genotyping of the OPRM1 gene rs1799971 variant associated with opioid use disorders
Source: BMC Res Notes. 2023 Nov 14;16:333. doi: 10.1186/s13104-023-06578-7 (PMC10648702; doi:10.1186/s13104-023-06578-7)
Supplement: Supplementary file 1 — Supplementary Material 1: Figure S1 - Gel bands of patient samples from 1 to 4. Figure S2 – Gel bands of patient samples from 5 to 21 with known A/G and G/G bands. Figure S3 – Gel bands of patient samples from 22 to 38 with known A/G and G/G bands. Figure S4 – Gel bands of patient samples from 39 to 52 with known A/G and G/G bands [file 13104_2023_6578_MOESM1_ESM.docx]

**Supplementary files**

Additional file 1: Figure S1, S2, S3 and S4. Gel band patterns of samples for *OPRM1* rs1777971 variant.

**
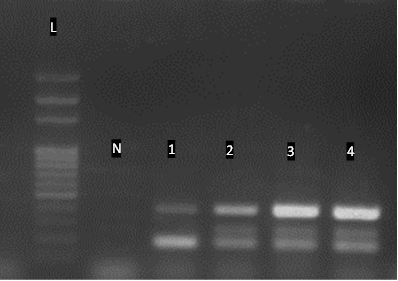
**

Figure S1 – Gel bands of patient samples from 1 to 4.

**
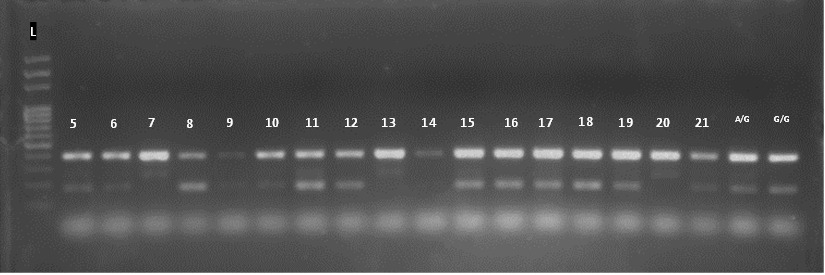
**

Figure S2 – Gel bands of patient samples from 5 to 21 with known A/G and G/G bands.

**
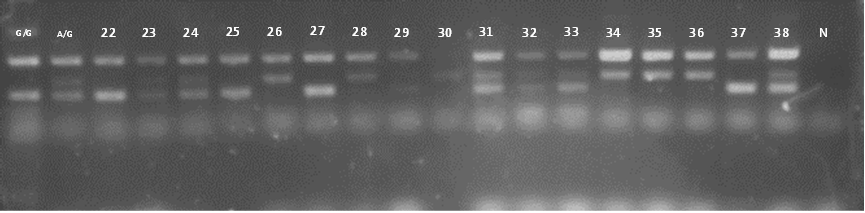
**

Figure S3 – Gel bands of patient samples from 22 to 38 with known A/G and G/G bands.


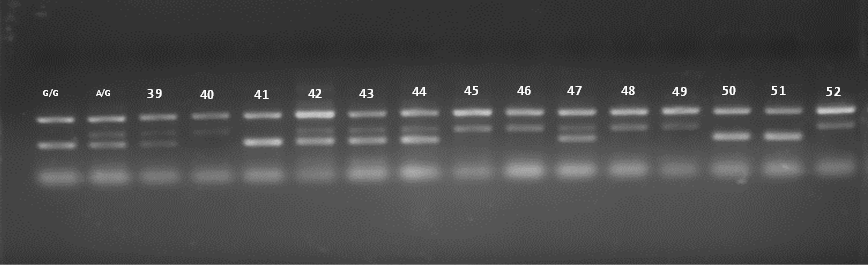
Figure S4 – Gel bands of patient samples from 39 to 52 with known A/G and G/G bands.
