# Supplementary material for: A novel tetra-primer ARMS-PCR for genotyping of the OPRM1 gene rs1799971 variant associated with opioid use disorders
Source: BMC Res Notes. 2023 Nov 14;16:333. doi: 10.1186/s13104-023-06578-7 (PMC10648702; doi:10.1186/s13104-023-06578-7)
Supplement: Supplementary file 2 — Supplementary Material 2: Table S1 - Genotyping results of the 52 volunteer patients. [file 13104_2023_6578_MOESM2_ESM.docx]

**Supplementary files**

Additional file 2: Table S1. Genotyping results of the 52 volunteer patients.

Table S1 – Genotype data of 52 patient samples

| **#** | **Patient Number** | **Sample ID** | **Genotype** | | |
| --- | --- | --- | --- | --- | --- |
|  |  |  | **A/A** | **A/G** | **G/G** |
| 1 | B56 | S1 |  | 1 |  |
| 2 | A41 | S2 |  | 1 |  |
| 3 | B40 | S3 |  | 1 |  |
| 4 | C14 | S4 |  |  | 1 |
| 5 | C29 | S5 |  |  | 1 |
| 6 | B61 | S6 |  |  | 1 |
| 7 | C14 | S7 | 1 |  |  |
| 8 | C22 | S8 |  |  | 1 |
| 9 | C38 | S9 |  |  | 1 |
| 10 | E02 | S10 |  |  | 1 |
| 11 | A75 | S11 |  | 1 |  |
| 12 | A45 | S12 |  |  | 1 |
| 13 | C11 | S13 | 1 |  |  |
| 14 | D03 | S14 |  |  | 1 |
| 15 | F10 | S15 |  |  | 1 |
| 16 | D09 | S16 |  |  | 1 |
| 17 | C34 | S17 |  |  | 1 |
| 18 | C07 | S18 |  |  | 1 |
| 19 | A40 | S19 |  |  | 1 |
| 20 | B59 | S20 | 1 |  |  |
| 21 | C17 | S21 |  |  | 1 |
| 22 | D4 | S22 |  |  | 1 |
| 23 | D13 | S23 |  | 1 |  |
| 24 | A57 | S24 |  | 1 |  |
| 25 | D14 | S25 |  |  | 1 |
| 26 | A77 | S26 | 1 |  |  |
| 27 | G3 | S27 |  |  | 1 |
| 28 | A44 | S28 | 1 |  |  |
| 29 | F16 | S29 |  |  | 1 |
| 30 | B60 | S30 | 1 |  |  |
| 31 | A52 | S31 |  | 1 |  |
| 32 | F22 | S32 |  | 1 |  |
| 33 | A50 | S33 |  | 1 |  |
| 34 | B55 | S34 | 1 |  |  |
| 35 | B64 | S35 | 1 |  |  |
| 36 | A51 | S36 | 1 |  |  |
| 37 | F11 | S37 |  |  | 1 |
| 38 | G2 | S38 |  | 1 |  |
| 39 | F13 | S39 |  | 1 |  |
| 40 | B21 | S40 | 1 |  |  |
| 41 | G1 | S41 |  |  | 1 |
| 42 | A81 | S42 |  | 1 |  |
| 43 | A74 | S43 |  | 1 |  |
| 44 | H2 | S44 |  | 1 |  |
| 45 | H1 | S45 | 1 |  |  |
| 46 | B63 | S46 | 1 |  |  |
| 47 | C16 | S47 |  | 1 |  |
| 48 | C19 | S48 | 1 |  |  |
| 49 | A55 | S49 | 1 |  |  |
| 50 | C28 | S50 |  |  | 1 |
| 51 | F30 | S51 |  |  | 1 |
| 52 | F24 | S52 | 1 |  |  |
|  |  |  |  |  |  |
|  | Total |  | 15 | 15 | 22 |
